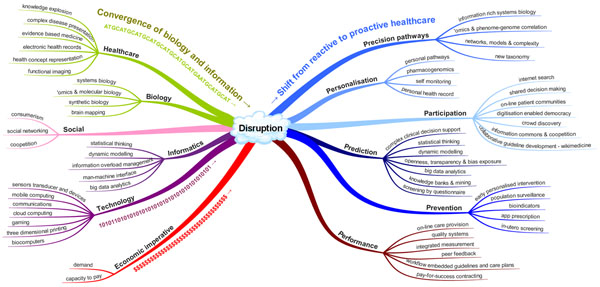# The role of informatics in the shift from reactive to proactive healthcare

**DOI:** 10.1186/1878-5085-5-S1-A50

**Published:** 2014-02-11

**Authors:** Michael Legg

**Affiliations:** 1 Michael Legg & Associates and Royal College of Pathologists of Australasia, Sydney Australia

## 

Just as John Snow used a branch of informatics, geospatial analysis, to identify the source of cholera in London and so prove the germ theory and revolutionise medicine in 1854, so too will the application of informatics and associated technology be instrumental to the next big change in healthcare. All countries with advanced economies are facing increased demand from ageing populations and increased chronic disease. Many also have shortages of skilled workers. Mounting evidence points to avoidable errors causing serious harm in health systems. Indeed optimal care only occurs about half the time in even the best performing health systems [1]. Doing more of what we do now just a little better, even if that is continuous, will not be enough to address the looming crisis in sustainable healthcare. Convergence in health care, biology, informatics and technology together with the associated social changes and economic imperative is driving a paradigmal shift [2] that may be the answer. Informatics has a role in most aspects of this. This paper examines that role. Figure 1 provides a summary. In terms of our understanding of physiological pathways, informatics is now the major tool of molecular biology with for example a 3 to 1 ratio in time spent computing versus chemistry for gene sequencing. Informatics is being used to map neural networks and to build the models of systems biology [3] with ever increasing levels of precision and complexity that simply can’t be done without the help of machines. Our understanding has changed so much recently that the American Academy of Science is now arguing that it is time for a new taxonomy [4]. Personalisation is occurring both because of social change and increased biological knowledge and is being facilitated by cheap mobile computing, sensors and devices. These social forces and the enabling technologies are allowing greater participation by ‘non-experts’ in decision making, treatment, discovery and knowledge management. Greater knowledge about how we think [5], advances in the information sciences and availability of computing power has meant our capacity to acquire knowledge and use it to predict the course of pathology has increased enormously and it is just as well because the explosion of information is impossible to deal with otherwise. These new methods can be used to provide better advice and to better prevent disease through discovery, monitoring and treatment. The health system itself can also benefit from what looks like a second phase of utilisation of information technology through on-line care provision, real integrated measurement of quality and integration of knowledge in work-flow. With openness and transparency there is also the prospect of competition and with good measures that pay-for-success contracting may be used as a positive lever. Large scale change in the way healthcare is done is both essential and inevitable. It is likely that this will derive from the merging of the knowledge and machines of the biological and information revolutions facilitating a shift from reactive treatment to proactive personalised medicine. Only by such significant phenomenon could the quantum improvement in the effectiveness and efficiency of healthcare which is needed come. Digitisation of biology and health will allow machines to help, lead to a demystification of disease, the democratisation of healthcare, and a move from the treatment of disease to the promotion and maintenance of wellness. The development of digital technology has disrupted other sectors notably media, retail and manufacturing. Medicine is unlikely to remain immune from this societal change [6].

**Figure 1:**